# Stand like an Anvil

**Published:** 1864-01

**Authors:** Bishop Doane


### STAND LIKE AN ANVIL.

BY BISHOP DOANE.

" Stand like an anvil!" when the strokes
Of stalwart strength fall thick and fast;
Storms but more deeply root the oaks,
Whose brawny arms embrace the blast.

" Stand like an anvil!" when the sparks
Fly far and wide, a fiery shower;
Virtue and truth must still be marks
Where malice proves its want of power.

" Stand like an anvil!" when the bar
Lies red and glowing on its breast;
Duty shall be life's leading star,
And conscious innocence its rest.

" Stand like an anvil!" when the sound
Of ponderous hammers pains the ear;
Thine but the still and stern rebound
Of the great heart that can not fear.

" Stand like an anvil!" noise and heat
Are born of earth, and die with time;
The soul, like God, its source and seat
Is solemn, still, serene, sublime.

